# The Location of the Pseudoautosomal Boundary in *Silene latifolia*

**DOI:** 10.3390/genes11060610

**Published:** 2020-05-31

**Authors:** Marc Krasovec, Yu Zhang, Dmitry A. Filatov

**Affiliations:** 1 Department of Plant Sciences, University of Oxford, Oxford OX1 3RB, UK; yu.zhang@plants.ox.ac.uk (Y.Z.); dmitry.filatov@plants.ox.ac.uk (D.A.F.); 2 School of Life Science, Hunan University of Science and Technology, Xiangtan 411201, China

**Keywords:** sex chromosome, *Silene latifolia*, pseudoautosomal region, recombination

## Abstract

Y-chromosomes contain a non-recombining region (NRY), and in many organisms it was shown that the NRY expanded over time. How and why the NRY expands remains unclear. Young sex chromosomes, where NRY expansion occurred recently or is on-going, offer an opportunity to study the causes of this process. Here, we used the plant *Silene latifolia*, where sex chromosomes evolved ~11 million years ago, to study the location of the boundary between the NRY and the recombining pseudoautosomal region (PAR). The previous work devoted to the NRY/PAR boundary in *S. latifolia* was based on a handful of genes with locations approximately known from the genetic map. Here, we report the analysis of 86 pseudoautosomal and sex-linked genes adjacent to the *S. latifolia* NRY/PAR boundary to establish the location of the boundary more precisely. We take advantage of the dense genetic map and polymorphism data from wild populations to identify 20 partially sex-linked genes located in the “fuzzy boundary”, that rarely recombines in male meiosis. Genes proximal to this fuzzy boundary show no evidence of recombination in males, while the genes distal to this partially-sex-linked region are actively recombining in males. Our results provide a more accurate location for the PAR boundary in *S. latifolia*, which will help to elucidate the causes of PAR boundary shifts leading to NRY expansion over time.

## 1. Introduction

Sex chromosomes are known to evolve from autosomes (e.g., [1,2]) following acquisition of sex-determining gene(s) and evolution of a non-recombining region around the sex locus (reviewed in [3,4]). Following formation of a non-recombining sex-determining region (SDR), a part of sex chromosomes continues to recombine in heterogametic sex, comprising the so-called pseudoautosomal region (PAR). As the PAR is partially sex-linked, its properties are intermediate between the sex chromosomes and the autosomes, but they also possess some features unique to these peculiar genomic regions [5]. In particular, the recombination rate may be unusually high in this region—e.g., in humans the average recombination rate in the p-arm PAR is at least 10 times higher than the genomic average [6,7], while local recombination in mouse PAR is 100 times higher than the genomic average [8]. This elevated recombination in the PAR is the consequence of X:Y pairing only in this region during male meiosis and the physical size of the region is negatively proportionate to recombination density. Frequent recombination in the PAR may inflate GC-content via biased gene conversion [9] and increase mutation rate in this region [10].

The genes in the PAR show a unique evolutionary dynamic specific to this region [5,11,12]. In particular, the PAR genes closely linked to the SDR are expected to maintain some sequence divergence between the X- and Y-linked alleles, which should inflate polymorphism in the PAR. Furthermore, the genes evolving under sex-specific or sexually antagonistic (SA) selection may accumulate divergence in frequencies of alleles linked to the X and Y chromosomes, which could favor recombination suppression and lead to shrinking of the PAR and expansion of the non-recombining SDR [5]. Indeed, the non-recombining SDRs, such as male-specific region on mammalian Y-chromosome (NRY) or female-specific region on bird W-chromosome (NRW), show a tendency to expand over evolutionary time. In human ancestry, this expansion has occurred in four [13] or five [14] steps, giving rise to so-called “evolutionary strata”, with the most recent expansion about 30 million years ago [14]. The analyses of bird W-chromosomes show evidence of multiple independent expansion events in different lineages [2]. NRY (or NRW) expansion has also been reported for other lineages, such as snakes [15], and dioecious plants including the Silene genus [16]. SA selection is often mentioned as the cause of this expansion [17,18], though there is relatively little experimental evidence supporting this view [19,20]. Furthermore, resolving SA does not have to involve NRY expansion; e.g., it can be resolved by limiting SA gene expression to one sex. The latter mechanism was suggested to be at play in ratite birds, such as emus, where the NRW is relatively small, while the extent of sex-biased expression in the pseudoautosomal genes is substantial [21], though it remains unclear whether these two observations are causally linked. Sex-biased expression evolved multiple times in eukaryotes and has been reported in other species such as in the plant *Silene latifolia* [22].

The proximate mechanisms that cause NRY expansion and recombination suppression between sex chromosomes are not well understood [19]. Such mechanisms could involve chromosomal rearrangements preventing recombination, or operate via regulation of rate and/or distribution of recombination in the genome. The latter type includes sex-specific achiasmy, when recombination occurs only in the homogametic sex, as found in Drosophila and butterflies [23], but may also include more subtle changes in local recombination rate in the region adjacent to the NRY/PAR boundary. Translocations of chromosomal segments from autosomes to the Y-chromosome, resulting in recombination suppression in the translocated region, can be regarded as an instance of the former mechanism. Such translocations can lead to formation of additional sex chromosomes, the co-called neo-sex chromosomes reported for many species, including *Drosophila miranda* (e.g., [24]) and the plant *Silene diclinis* [25]. In some cases, this process of translocation leading to the formation of neo-sex chromosomes has been repeated multiple times. This is particularly the case in monotremes, where this process resulted in formation of five X- and five Y-chromosomes in platypus [26]. In addition, chromosomal inversion(s) may also play a significant role in sex chromosome evolution and NRY expansion [19], such as reported in papaya [27] and sticklebacks [28]. However, in some cases, NRY expansions appear to have occurred without inversions involved [29]. Moreover, inversions detectable in NRY regions could be the consequence rather than the cause of recombination suppression, as they can occur between the sequences present in multiple copies, such as transposable elements that tend to accumulate in the non-recombining regions [30,31]. The analysis of genes in the region adjacent to the PAR boundary (e.g., [8,32,33]) is essential to understand the mechanisms underpinning NRY expansion and shifts in PAR boundary location.

The studies of recently evolved sex (or neo-sex) chromosomes have contributed significantly to our understanding of sex chromosome evolution, notably in plants [34], such as found in *Silene latifolia* and its close relatives [35]. They represent convenient study systems to investigate the processes shaping sex chromosome at the early stage of their evolution [36,37]. In particular, the *S. latifolia* sex chromosomes have been actively used to study many aspects of sex chromosome evolution, ranging from the origin of sex chromosomes evolving de novo [1], to sex chromosome structure [38,39], to Y-degeneration [40,41,42,43,44], to evolution of dosage compensation [45,46,47,48], to NRY expansion [16,33,49,50,51]. In particular, it has been demonstrated that NRY expansion has created distinct evolutionary strata on *S. latifolia* sex chromosomes [16,52] that are analogous to evolutionary strata described on sex chromosomes of humans [13] and other species [15,53]. Furthermore, there is some evidence that the NRY expansion in *S. latifolia* is an on-going gradual process, as the NRY/PAR boundary in *S. latifolia* is “fuzzy” [49] and its location differs between close relatives of *S. latifolia* [33]. The previous work on this system was limited to a relatively small number of genetic markers in the PAR and the adjacent region. In this paper, we report the analysis of 86 genes adjacent to the PAR boundary, which allows us to substantially improve resolution in this region. A more accurate location of the PAR boundary reported by our study will significantly facilitate the downstream work devoted to the analysis of the processes and mechanisms involved in NRY expansion.

## 2. Materials and Methods 

### 2.1. Finding the Markers Common with Other Studies

The sequences of sex-linked and pseudoautosomal genes mapped previously [47] were blast-searched against the markers of the other study that analyzed the location of the PAR boundary [49] with blastall v2.2.26 (-p blastn) to identify the markers common to the two studies. In an attempt to increase the number of common markers, we also blast-searched the markers of each of these studies against the partial genome assemblies published previously [41,47]. In all cases, we kept blast hits with a *P*-value below 1.0 × 10^−80^ and identity higher than 97.5%. 

### 2.2. Finding the Location of the PAR/NRY Boundary

To identify Y-linked alleles in the genes with gametologs on the X and Y-chromosomes, we used transcriptome sequencing data from parents and 52 progeny (20 males and 32 females) of *S. latifolia* genetic cross df108 [47]. Y-linked alleles were identified as alleles always inherited from father to sons across two generations. To test whether occasional recombination occurs in the sex-linked genes located closely to the PAR boundary, we checked the presence of these Y-linked alleles in wild *S. latifolia* females sampled around Europe (5 females and 3 males, Table 1), with the expectation that fully Y-linked alleles are never present in the females. 

Plants used for RNA extraction and transcriptome sequencing were grown in the glasshouse (16h light, ambient temperature) from wild-collected seeds. Total RNA was extracted from young actively growing leaves using the Qiagen RNeasy Plant Mini kit (Qiagen, Manchester, UK) with the optional DNase digestions step, following the manufacturer’s instructions. RNA was poly-A enriched and sequenced on Illumina HiSeq 2000 at the WTCHG genomics facility in Oxford (UK). The newly generated sequence data is available from NCBI under bioproject number PRJNA629313 (biosamples accessions SAMN14776665 and SAMN14776666). Raw sequence reads were aligned against the reference transcriptome [47] with RSEM v.1.2.31 [54], bam files processed with Samtools v.1.2.1 [55] and single nucleotide polymorphism (SNP) calling carried out with HaplotypeCaller from GATK v. 4.1.2.0 [56]. Then, resulting VCF files for separate samples were merged together with bcftools v1.2 to calculate population genomics statistics (F_st_ between females and males and π calculated for two genders separately) with vcftools v0.1.15. We calculated the statistics for each gene with window size corresponding to the gene length and by removing indels (options: -chr, -remove-indels, -window-pi and -fst-window-size). Last, we generated the fasta files for each wild individual with FastaAlternateReferenceMaker (from GATK v. 4.1.2.0) and aligned the gene sequences with muscle v3.8.31 [57] to calculate Tajima’s D [58] with mstatspop v.0.1 [59]. The fasta alignments for the genes analyzed are available in Supplementary Materials.

## 3. Results

### 3.1. Finding the Markers in Common Between X-maps of Different Studies

The PAR and NRY/PAR boundary in *S. latifolia* have been actively studied using genetic mapping and population genetic approaches [33,49,50,51]. This paper takes advantage of a larger number of X-linked and pseudoautosomal genes in the genetic map we published previously [47] to more accurately locate the PAR boundary and test how wide the “fuzzy” boundary region is. We started by finding the markers in common between our map [47] and the most detailed map delimiting the *S. latifolia* PAR published by others (Figure 1 in [49]). Blast-searching the sequences of the two studies against each other identified only eight markers in common between the two maps of *S. latifolia* X-chromosome (shown in bold in Table 2). In order to achieve better integration between the mapping results of different studies, we used previously published genomic assembly [47] to identify the genomic contigs that contained genetic markers from different studies. This allowed us to add additional six markers corresponding to nearly identical genomic position, though over half of the markers from Qiu et al. [49] could not be found (Table 2), which is not too surprising given the published genomic assembly covers only a fraction of large (~3 Gb) *S. latifolia* genome [47].

The markers in common between the X-chromosome maps of [49] and [47] show very good correspondence in marker order (Table 2). The location of the PAR border differed slightly between the maps, with marker contig8488 designated as fully sex-linked by Papadopulos et al. [47], while Qiu et al. [49] concluded that the corresponding marker E780X is pseudoautosomal. Both studies agree that the *S. latifolia* PAR boundary is located somewhere more distally to the marker E779X/contig675. Thus, we focused our analyses on the genes between the sex-linked marker E779X/contig675 and the pseudoautosomal E241/contig3920.

### 3.2. Finding the Location of the PAR Boundary

The region between contig675 and contig3920 contains 86 genes in the previously published genetic map [47]. According to this map, the PAR boundary is located between the genes encoding transcripts contig9011 and contig16617 (Table 3) with the former being sex-linked and the latter being pseudoautosomal [47]. While segregation analysis of markers in the genetic cross is informative about the approximate location of the PAR boundary, it is unlikely to detect rare recombination events that may occur proximally to the putative PAR boundary. However, such rare events may be detected in the analysis of sequence polymorphism data from wild populations because such data contain information about multiple meioses that occurred since the common ancestor of the alleles in the sample. 

In order to look for such rare recombination events, we searched for “Y-SNPs”—Y-alleles identified by a segregation pattern in the df108 mapping family [47], in transcriptome data of five wild females (Table 1). As no Y-alleles were found in any of the genes designated as pseudoautosomal by [47], we focused this analysis on 48 genes located proximally to contig16617 (Table 3) in the genetic map from [47]. Out of these 48 genes, Y-alleles were found for 19 genes (Table 3 and Table S1). Six out of these 19 genes showed the presence of Y-alleles in some of the wild female samples (Table 3 and Table S1), indicating occasional recombination in male meiosis in these genes. Interestingly, most of the genes showing evidence for recombination in male meiosis are adjacent to the PAR boundary, while no such recombining genes were detected more proximally along the X-chromosome region analyzed (Table 3). Furthermore, the genes next to the PAR (between contigs 6406 and 1798) contained multiple Y-SNPs in several females, indicating that recombination in male meiosis is not too rare in this region. On the other hand, the genes proximally to contig6406 (mapped to 63 cM [47]) contained zero or one Y-SNPs in females (Table 3 and Table S1). Thus, contig6406 may represent a boundary between regions of relatively frequent and very rare recombination in male meiosis. For convenience, we refer to the partially sex-linked region between contigs 8558 and 16617 as a “fuzzy boundary” between the PAR and fully sex-linked genes and the region proximally and distally to contig6406 as fuzzy boundary I and II, respectively (Table 3).

### 3.3. Patterns of Genetic Diversity Around the PAR Boundary

The patterns of polymorphism, summarized by such statistics as average per nucleotide heterozygosity (π) difference between males and females, or population differentiation (F_st_) between the two sexes, are informative about the recombination between the partially X- and Y-linked alleles in males (e.g., [33,49]). In particular, in the absence of recombination, divergence between the X- and Y-linked alleles of a sex-linked gene inflates heterozygosity in males, but not in the females, so the difference in π between males and females indicates lack of recombination in a gene in males. Similarly, F_st_ can be used to measure “population differentiation” between males and females that is expected to be high in the absence of recombination in male meiosis and low if recombination is present.

**Figure 1:**
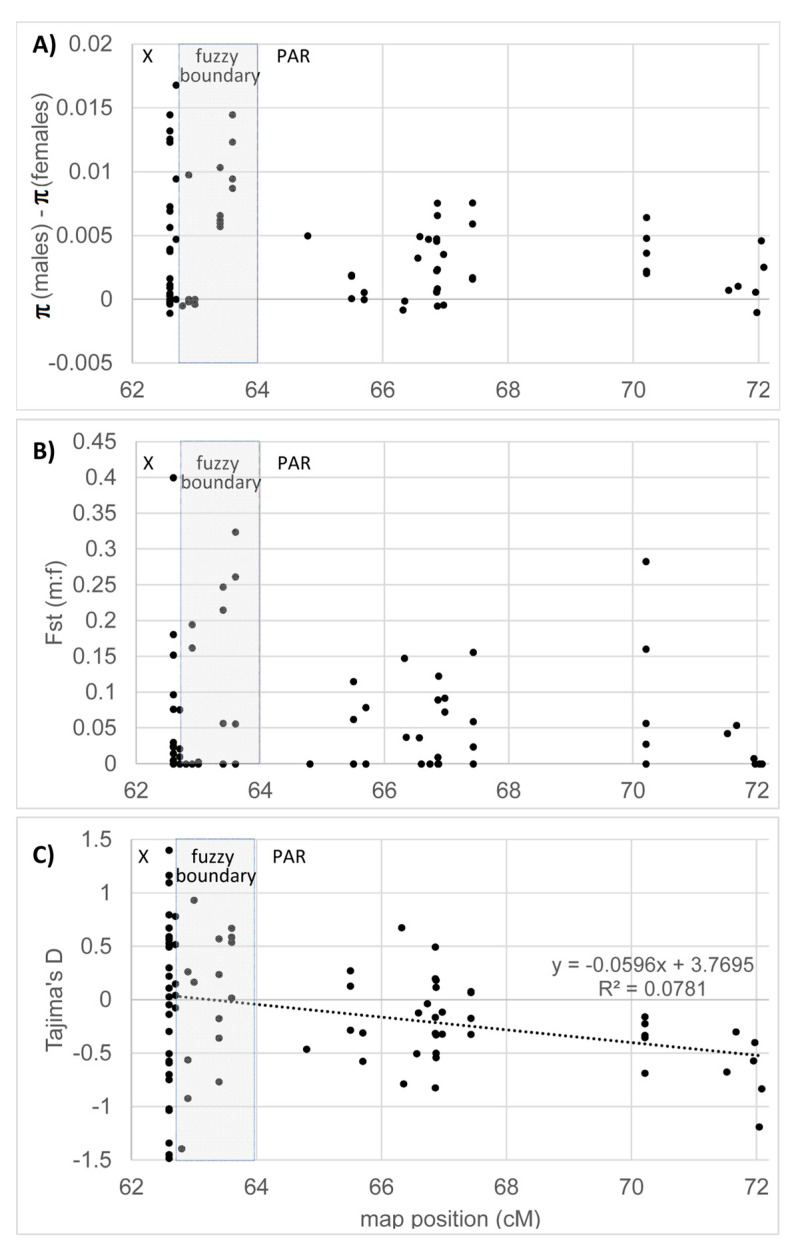
Patterns of DNA polymorphism in *S. latifolia* males and females at the genes adjacent to the PAR boundary. (**A**) The difference in average heterozygosity (π) between males and females; (**B**) Population differentiation (F_st_) between males and females and (**C**) Tajima’s D for the entire sample.

The population analysis using eight wild individuals (Table 1) focused on 86 genes (Table 3) from the previously published map [47], including the region from the fully sex-linked gene E779X/contig675 to the pseudoautosomal gene E241/contig3920. The overall genetic diversity shows contrasting patterns between the X-linked and pseudoautosomal genes and between the males and females (Figure 1A). The polymorphism is higher in males than females but the difference varies between the genes. On average, the polymorphisms in males and females for the PAR genes (distal to contig9011) are π_males_ = 0.0051 and π_females_ = 0.0025 (Student *t*-test, *p*-value < 0.01, with π_males_/π_females_ = 2.02). In the fuzzy boundary genes (from contigs 1798 to 9011), π_males_ = 0.0105 and π_females_ = 0.0025 (Student *t*-test, *p*-value < 0.01, with π_males_/π_females_ = 4.22), indicating that recombination in male meiosis is sufficiently rare in this fuzzy boundary region for the Y- and X-linked gametologs to accumulate significant sequence divergence. Last, the X-linked genes (proximal to contig1798) have π_males_ = 0.0064 and π_females_ = 0.0017 (Student *t*-test, *p*-value < 0.01, with π_males_/π_females_ = 3.72). F_st_ between males and females also sharply rises proximally to 64.0 cM map position (Figure 1B). Tajima’s D [58] is variable among the genes analyzed with an increase for genes in the sex-linked (Tajima’s D average of 0.059) and the fuzzy boundary regions (Tajima’s D average of 0.087) compared to the PAR (Tajima’s D average of −0.268) (Figure 1C). In the pseudoautosomal genes (distally to contig9011) Tajima’s D shows significant decline with the distance from the PAR boundary (linear regression model, *p*-value = 0.00915, Figure 1C).

### 3.4. Integration of Genetic Map and Genome Sequence for the PAR Boundary Region

In order to estimate physical size of the PAR and the fuzzy PAR boundary region we used the sequences of the sequenced transcripts encoded by genes adjacent to the PAR boundary to find the corresponding genomic scaffolds in the partial *S. latifolia* genome assembly published previously [47]. BLAST searches with stringent parameters (see methods) identified corresponding genomic scaffolds for 76 out of 86 genes in the proximity of the PAR boundary (Table S2). In total we identified 72 genomic scaffolds with the total length 2.84 Mb. Most genes analyzed corresponded to separate scaffolds, reflecting a highly fragmented state of the genome assembly. The only two exceptions to this were scaffolds QBIE01000100.1 and QBIE01001489.1 that contained four and two genes, respectively (shown in bold in Table S2). Reassuringly, the genes corresponding to the same scaffold are located closely in the genetic map (<1.9 cM apart), though not always adjacent to each other (Table S2). The total length of genomic scaffolds corresponding to markers in the PAR, fuzzy boundary and the sex-linked region adjacent to PAR boundary are 1.57 Mb, 0.49 Mb and 1.2 Mb, respectively (Table S2). However, given the fragmented state of the genomic assembly, these numbers represent gross underestimates of the actual physical size of these genomic regions. 

## 4. Discussion

Here, we integrated the data from genetic mapping, genome sequencing and population genetic analyses to establish the location of the boundary between the pseudoautosomal region and the X-chromosome in *S. latifolia*. There are many reasons that make PAR boundary region particularly interesting for evolutionary and molecular genetic studies [5,62]. The genes proximally to PAR boundary do not recombine in male meiosis, while the genes located distally, in the pseudoautosomal region, do recombine and the recombination rate may be unusually high [8]. How such dramatic difference in the recombination rate in the adjacent regions is determined at the molecular and chromosomal level is not entirely clear. Furthermore, the recombination (or lack of it) affects many evolutionary processes that shape the genome [63,64,65], and PAR boundary regions provide an interesting comparison between the “deserts” and “jungles” (cold- and hotspots) of recombination next to each other. Finally, the shifts of the PAR boundary are thought to play central role in evolution of the non-recombining region on the sex chromosomes [3,19].

Despite the intriguing evolutionary and molecular genetic aspects of the PAR boundary [5], its location is known only for a few species, including humans [66,67], some mammals [68,69,70,71], birds [2] and plants [32,49]. Many of these studies reported the changes in the location of the PAR boundary between closely related species [33,69], or even within a species [8,32,49], indicating that the location of the PAR boundary is often unstable and evolutionary labile. In particular, for *S. latifolia* the PAR boundary was reported to be “fuzzy” [49], implying that there is a region between the PAR and the sex-linked region where recombination in male meiosis occurs only rarely, and our results are consistent with this. The fuzzy boundary may represent a region of on-going recombination suppression leading to gradual expansion of the NRY. However, shifts of the PAR boundary could also lead to the opposite—an expansion of the PAR and shrinking of the sex-linked region, as was reported for *Mus spretus*, where a previously fully sex-linked 400 kb region adjacent to the PAR became pseudoautosomal [8,69]. Regardless of the direction of evolutionary change, the PAR boundary region provides an extremely interesting location in the genome to study evolution of recombination, which is key to our understanding of sex chromosome evolution and NRY formation.

In this paper, we expanded the number of genes in the proximity of the PAR boundary analyzed for the presence of recombination in male meiosis and patterns of genetic diversity. Instead of using a conventional genetic mapping approach that would fail to detect rare male recombination in the region adjacent to the PAR boundary, we opted to focus on the detection of Y-alleles in wild females. The latter approach effectively integrates over many thousands of meioses in the history of the sample and thus has a lot more power to detect rare recombination in males compared to conventional genetic mapping. Indeed, this analysis detected male recombination in six NRY genes located near the PAR boundary, but not in the genes further away from the PAR. These results indicate that the PAR boundary is located between the genes contig8598 (62.6 cM) and contig16617 (64.8 cM), with the former being fully sex-linked and the latter fully pseudoautosomal, with no male-specific alleles detectable and evidence of recombination in male meiosis in the genetic cross df108 [47]. The region between these markers must be partially sex-linked, as it shows intermediate properties between the fully sex-linked and pseudoautosomal genes. In particular, the genes in this partially sex-linked region show full sex-linkage in the genetic cross df108, and also show evidence of occasional recombination in male meiosis as some of the wild females contain the alleles that should be Y-linked based on segregation in df108 family. Interestingly, the genes proximally to contig6406 (mapped to 63 cM [47]) contained zero or one Y-SNPs in females (Table 3 and Table S1), perhaps because of lower recombination rate compared to the genes from 63.4 to 63.6 cM, or because of the action of gene conversion between the X- and Y-linked gametologs.

To compare our results with the previous work [33,44,49,50,51], we identified 14 markers (listed in Table 2) in common between our dataset and the markers in the most detailed map delimiting the PAR published by others (Figure 1 in [49]). The comparison of the markers reveals that the results of this study support the conclusion of [49] that PAR boundary is located more proximally compared to what had been concluded by [47]. The results of the latter study are based entirely on segregation in a genetic cross and the aim of that study was not to precisely locate the *S. latifolia* PAR boundary. On the other hand, both the current paper and the study published by Qiu et al. [49] used additional analyses based on DNA polymorphism in wild populations, which allowed these studies to identify rare recombination events undetectable in a genetic cross. We improved on the results of [49] by adding substantially more genes in the vicinity of the *S. latifolia* PAR boundary, helping to locate the PAR boundary more accurately and facilitating further studies on the evolution of on-going recombination suppression and NRY expansion. For example, it will be particularly interesting to study chromatin structure in this partially sex-linked region and compare it with the chromatin in the fully sex-linked region and the PAR.

The results of polymorphism analyses are consistent with the PAR boundary located proximally to contig16617. Most genes proximally to contig16617 show much higher genetic diversity in males compared to females, which indicates some degree of divergence between the X- and Y-linked gametologs that contributes to polymorphism in males. Interestingly, this is true even for the presumably pseudoautosomal genes between contigs 16617 and 8598 where Y-alleles are occasionally found in females, indicating that recombination in male meiosis is sufficiently rare in this fuzzy boundary region for the Y- and X-linked gametologs to accumulate sequence divergence. 

The patterns of polymorphism in the pseudoautosomal genes are consistent with balancing selection maintaining excess of intermediate frequency polymorphisms at the genes in the vicinity of the NRY/PAR-boundary. This is evidenced by elevated F_st_ and the difference in polymorphism in males and females, as well as the negative regression of Tajima’s D with distance from the NRY/PAR-boundary (Figure 1C). The latter result is consistent with the previous report of inflated Tajima’s D in pseudoautosomal genes adjacent to the PAR-boundary [49]. However, that study reported positive Tajima’s D only in the two genes immediately adjacent to the PAR boundary, E559 and cs3297, while more distally located genes between E523 and E241 showed negative Tajima’s D (Figure 2B in [49]). Although we found no homologs for the markers E559 and cs3297 in our dataset, they are likely to be located in the fuzzy boundary region (proximally to contig16617) rather than in the PAR (Table 2). Our analysis also showed that many genes in the fuzzy boundary region have positive Tajima’s D and for more distal PAR genes Tajima’s D is lower and becomes negative closer to E241/contig3920 (Figure 1C). It is possible that this pattern is caused by linkage to the NRY and the presence of sexually antagonistic genes partially linked to NRY, although, according to theory, this is likely only for pseudoautosomal genes located very close to the PAR boundary [12,18,72] and most PAR genes analyzed in our study are likely located too far for linkage with NRY to affect the patterns of polymorphism. 

The lack of adequate genome sequence assembly for *S. latifolia* genome generally, and for the PAR boundary region specifically, remains a significant limitation of what could be carried out next in this species. In particular, nearly all genes analyzed in this study fall into separate genomic scaffolds, reflecting the highly fragmented state of the assembly that does not allow us to even approximately estimate the physical sizes of the fully sex-linked, partially sex-linked and pseudoautosomal regions of the X-chromosome. The total lengths of the genomic scaffolds corresponding to genes mapped to these regions can serve only as a lower boundary. Nevertheless, it is clear that the PAR should comprise a significant proportion of *S. latifolia* X-chromosome because a third of the genes mapped to that chromosome are pseudoautosomal (108 out of 327, [47]). Our results demonstrate that at least 20 more genes previously designated as sex-linked [47] are actually pseudoautosomal (or only partly sex-linked), increasing the proportion of the PAR genes further. The physical size of this partially sex-linked fuzzy boundary region must be substantial, as even the minimal estimate, provided by the total length of genomic scaffolds in this region, is nearly half a megabase (Table S2), which is likely to be a gross underestimation of the actual size of this region. Better, more contiguous assembly for the *S. latifolia* genome is long overdue and will significantly advance the analysis of sex chromosome structure and evolution in this interesting plant model species.

## Figures and Tables

**Table 1:** *S. latifolia* samples, for which transcriptome sequence data were analyzed in this study.

ID	Sex	Country	Location	Data from
Sa283g	male	Belgium		[60]
Sa668a	male	Sweden	Oland	[60]
Sa984	male	England		[47]
Sa526b	female	Austria	Stift Melk	[61]
Sa615	female	Germany		This study
Sa758d	female	Russian Federation	Moscow	This study
Sa833d	female	Spain		[43]
Sa985	female	Austria		[47]

**Table 2:** The markers in common between different studies of the pseudoautosomal region (PAR) and the X chromosome. The markers in bold are the same genes in both maps; the other markers (not in bold) co-locate on genomic scaffolds, but they are not the same genes.

Qui et al. 2016 [49]			Papadopoulos et al. 2015 [47]		
Marker	Map (cM)	X or PAR	Marker	Map (cM)	X or PAR
**E707X**	0	X	**Contig4232**	4.3	X
**SlX4**	4.2	X	**Contig8519**	14.5	X
SIX6A	5.4	X	Contig14178	13.4	X
**SlX7**	7.7	X	**Contig842**	18.2	X
E711X	14.1	X	none	-	-
SlX3	28.1	X	none	-	-
E713X	36.3	X	Contig19016	35.7	X
E807X	44.5	X	none	-	-
E330X	60.5	X	none	-	-
**SlCypX**	67.9	X	**Contig8805**	52.6	X
SIX9	67.9	X	none	-	-
**E777X**	71.4	X	**Contig3001**	60.9	X
E779X	71.4	X	Contig675	62.6	X
cs1536X	81.5	X	none	-	-
E799X	82.6	X	none	-	-
cs3597	83.8	PAR	none	-	-
**E780X**	83.8	PAR	**Contig8488**	62.7	X
E316X	83.8	PAR	none	-	-
E559X	83.8	PAR	none	-	-
E521X	83.8	PAR	none	-	-
E523X	83.8	PAR	none	-	-
cs32X	84.9	PAR	Contig16105	65.7	PAR
E247X	84.9	PAR	none	-	-
SlX6B	84.9	PAR	none	-	-
SlCyt	84.9	PAR	none	-	-
E200	86.1	PAR	none	-	-
cs1539	86.1	PAR	none	-	-
**E241**	86.1	PAR	**Contig3920**	72.1	PAR
**cs4991**	86.1	PAR	**Contig7492**	76.2	PAR
E352X	85.5	PAR	Contig4019	80.6	PAR
E592	101.3	PAR	Contig11138	89.7	PAR
cs5136X	120.6	PAR	none	-	-

**Table 3:** The presence of Y-SNPs in wild females (genotypes are listed in Table S1).

Genes	Map Position	df108 Map	This Study	Y-SNPs		
				Analyzed	In Females	Females with Y-SNPs
Contig675	62.6	X	X	0	-	-
Contig697	62.6	X	X	5	0	0
Contig804	62.6	X	X	0	-	-
Contig867	62.6	X	X	0	-	-
Contig8509	62.6	X	X	0	-	-
Contig8660	62.6	X	X	0	-	-
Contig15301	62.6	X	X	1	0	0
Contig1564	62.6	X	X	0	-	-
Contig1740	62.6	X	X	0	-	-
Contig17645	62.6	X	X	0	-	-
Contig18491	62.6	X	X	0	-	-
Contig1290	62.6	X	X	0	-	-
Contig1436	62.6	X	X	0	-	-
Contig15401	62.6	X	X	0	-	-
Contig12513	62.6	X	X	3	0	0
Contig1804	62.6	X	X	0	-	-
Contig18911	62.6	X	X	0	-	-
Contig2431	62.6	X	X	0	-	-
Contig2761	62.6	X	X	0	-	-
Contig2802	62.6	X	X	0	-	-
Contig3835	62.6	X	X	0	-	-
Contig14349	62.6	X	X	17	0	0
Contig3846	62.6	X	X	0	-	-
Contig4210	62.6	X	X	0	-	-
Contig4518	62.6	X	X	0	-	-
Contig17773	62.6	X	X	27	0	0
Contig5724	62.6	X	X	0	-	-
Contig8598	62.6	X	X	11	0	0
Contig1798	62.7	X	fuzzy boundary I	4	1	1
Contig8488	62.7	X	fuzzy boundary I	0	-	-
Contig9505	62.7	X	fuzzy boundary I	14	1	3
Contig18786	62.7	X	fuzzy boundary I	2	0	0
Contig255	62.7	X	fuzzy boundary I	0	-	-
Contig12476	62.8	X	fuzzy boundary I	0	-	-
Contig2117	62.9	X	fuzzy boundary I	1	0	0
Contig1858	62.9	X	fuzzy boundary I	0	-	-
Contig456	62.9	X	fuzzy boundary I	0	-	-
Contig1229	63.0	X	fuzzy boundary I	0	-	-
Contig6406	63.0	X	fuzzy boundary I	0	-	-
Contig1046	63.4	X	fuzzy boundary II	13	4	3
Contig1251	63.4	X	fuzzy boundary II	24	4	3
Contig13504	63.4	X	fuzzy boundary II	5	0	0
Contig1623	63.4	X	fuzzy boundary II	3	0	0
Contig528	63.4	X	fuzzy boundary II	11	0	0
Contig13419	63.6	X	fuzzy boundary II	23	4	2
Contig15757	63.6	X	fuzzy boundary II	13	0	0
Contig15519	63.6	X	fuzzy boundary II	2	0	0
Contig9011	63.6	X	fuzzy boundary II	1	1	3
Contig16617	64.8	PAR	PAR	0	-	-
36 genes	-	PAR	PAR	0	-	-
Contig3920	72.1	PAR	PAR	0	-	-

## Supplementary Materials

The following are available online at https://www.mdpi.com/2073-4425/11/6/610/s1, Table S1. Genotype of wild females at SNPs inherited from father to sons in the df108 family [47]; Table S2. Location of genes adjacent to PAR-boundary in the *S. latifolia* genomic scaffolds published by Papadopulos et al. 2015 [47].

## References

[1] Filatov D.A. (2005). Evolutionary history of *Silene latifolia* sex chromosomes revealed by genetic mapping of four genes. Genetics.

[2] Zhou Q., Zhang J., Bachtrog D., An N., Huang Q., Jarvis E.D., Gilbert M.T.P., Zhang G. (2014). Complex evolutionary trajectories of sex chromosomes across bird taxa. Science.

[3] Charlesworth D. (2017). Evolution of recombination rates between sex chromosomes. Philos. Trans. R Soc. Lond. B Biol. Sci..

[4] Charlesworth D., Pagel M., Pomiankowski A. (2008). Sex chromosome origins and evolution. Evolutionary Genomics and Proteomics.

[5] Otto S.P., Pannell J.R., Peichel C.L., Ashman T.L., Charlesworth D., Chippindale A.K., Delph L.F., Guerrero R.F., Scarpino S.V., McAllister B.F. (2011). About PAR: The distinct evolutionary dynamics of the pseudoautosomal region. Trends Genet..

[6] Lien S., Szyda J., Schechinger B., Rappold G., Arnheim N. (2000). Evidence for heterogeneity in recombination in the human pseudoautosomal region: High resolution analysis by sperm typing and radiation-hybrid mapping. Am. J. Hum. Genet..

[7] Yu A., Zhao C., Fan Y., Jang W., Mungall A.J., Deloukas P., Olsen A., Doggett N.A., Ghebranious N., Broman K.W. (2001). Comparison of human genetic and sequence-based physical maps. Nature.

[8] Morgan A.P., Bell T.A., Crowley J.J., Pardo-Manuel de Villena F. (2019). Instability of the pseudoautosomal boundary in house mice. Genetics.

[9] Marais G. (2003). Biased gene conversion: Implications for genome and sex evolution. Trends Genet..

[10] Filatov D.A., Gerrard D.T. (2003). High mutation rates in human and ape pseudoautosomal genes. Gene.

[11] Kirkpatrick M., Guerrero R.F., Scarpino S.V. (2010). Patterns of neutral genetic variation on recombining sex chromosomes. Genetics.

[12] Charlesworth B., Jordan C.Y., Charlesworth D. (2014). The evolutionary dynamics of sexually antagonistic mutations in pseudoautosomal regions of sex chromosomes. Evolution.

[13] Lahn B.T., Page D.C. (1999). Four evolutionary strata on the human X chromosome. Science.

[14] Hughes J.F., Skaletsky H., Brown L.G., Pyntikova T., Graves T., Fulton R.S., Dugan S., Ding Y., Buhay C.J., Kremitzki C. (2012). Strict evolutionary conservation followed rapid gene loss on human and rhesus Y chromosomes. Nature.

[15] Vicoso B., Emerson J.J., Zektser Y., Mahajan S., Bachtrog D. (2013). Comparative sex chromosome genomics in snakes: Differentiation, evolutionary strata, and lack of global dosage compensation. PLoS Biol..

[16] Nicolas M., Marais G., Hykelova V., Janousek B., Laporte V., Vyskot B., Mouchiroud D., Negrutiu I., Charlesworth D., Moneger F. (2005). A gradual process of recombination restriction in the evolutionary history of the sex chromosomes in dioecious plants. PLoS Biol..

[17] Rice W.R. (1987). The accumulation of sexually antagonistic genes as a selective agent promoting the evolution of reduced recombination between primitive sex chromosomes. Evolution.

[18] Jordan C.Y., Charlesworth D. (2012). The potential for sexually antagonistic polymorphism in different genome regions. Evolution.

[19] Bergero R., Charlesworth D. (2009). The evolution of restricted recombination in sex chromosomes. Trends Ecol. Evol..

[20] Ironside J.E. (2010). No amicable divorce? Challenging the notion that sexual antagonism drives sex chromosome evolution. Bioessays.

[21] Vicoso B., Kaiser V.B., Bachtrog D. (2013). Sex-biased gene expression at homomorphic sex chromosomes in emus and its implication for sex chromosome evolution. Proc. Natl. Acad. Sci. USA.

[22] Zemp N., Tavares R., Muyle A., Charlesworth D., Marais G.A., Widmer A. (2016). Evolution of sex-biased gene expression in a dioecious plant. Nat. Plants.

[23] Bull J.J. (1983). Evolution of Sex Determining Mechanisms.

[24] Marion de Proce S., Halligan D.L., Keightley P.D., Charlesworth B. (2009). Patterns of DNA-sequence divergence between *Drosophila miranda* and *D. pseudoobscura*. J. Mol. Evol..

[25] Howell E.C., Armstrong S.J., Filatov D.A. (2009). Evolution of neo-sex chromosomes in *Silene diclinis*. Genetics.

[26] Rens W., Grutzner F., O’Brien P.C., Fairclough H., Graves J.A., Ferguson-Smith M.A. (2004). Resolution and evolution of the duck-billed platypus karyotype with an X1Y1X2Y2X3Y3X4Y4X5Y5 male sex chromosome constitution. Proc. Natl. Acad. Sci. USA.

[27] Wang J., Na J.K., Yu Q., Gschwend A.R., Han J., Zeng F., Aryal R., VanBuren R., Murray J.E., Zhang W. (2012). Sequencing papaya X and Yh chromosomes reveals molecular basis of incipient sex chromosome evolution. Proc. Natl. Acad. Sci. USA.

[28] Roesti M., Hendry A.P., Salzburger W., Berner D. (2012). Genome divergence during evolutionary diversification as revealed in replicate lake-stream stickleback population pairs. Mol. Ecol..

[29] Sun Y., Svedberg J., Hiltunen M., Corcoran P., Johannesson H. (2017). Large-scale suppression of recombination predates genomic rearrangements in *Neurospora tetrasperma*. Nat. Commun..

[30] Rozen S., Skaletsky H., Marszalek J.D., Minx P.J., Cordum H.S., Waterston R.H., Wilson R.K., Page D.C. (2003). Abundant gene conversion between arms of palindromes in human and ape Y chromosomes. Nature.

[31] Charlesworth B., Sniegowski P., Stephan W. (1994). The evolutionary dynamics of repetitive DNA in eukaryotes. Nature.

[32] Lappin F.M., Medert C.M., Hawkins K.K., Mardonovich S., Wu M., Moore R.C. (2015). A polymorphic pseudoautosomal boundary in the *Carica papaya* sex chromosomes. Mol. Genet. Genomics.

[33] Campos J.L., Qiu S., Guirao-Rico S., Bergero R., Charlesworth D. (2017). Recombination changes at the boundaries of fully and partially sex-linked regions between closely related Silene species pairs. Heredity (Edinb).

[34] Charlesworth D. (2016). Plant sex chromosomes. Annu. Rev. Plant Biol..

[35] Bernasconi G., Antonovics J., Biere A., Charlesworth D., Delph L.F., Filatov D., Giraud T., Hood M.E., Marais G.A., McCauley D. (2009). Silene as a model system in ecology and evolution. Heredity (Edinb).

[36] Charlesworth D. (2015). Plant contributions to our understanding of sex chromosome evolution. New Phytol..

[37] Muyle A., Shearn R., Marais G.A. (2017). The evolution of sex chromosomes and dosage compensation in plants. Genome Biol. Evol..

[38] Kazama Y., Ishii K., Aonuma W., Ikeda T., Kawamoto H., Koizumi A., Filatov D.A., Chibalina M., Bergero R., Charlesworth D. (2016). A new physical mapping approach refines the sex-determining gene positions on the *Silene latifolia* Y-chromosome. Sci. Rep..

[39] Armstrong S.J., Filatov D.A. (2008). A cytogenetic view of sex chromosome evolution in plants. Cytogenet. Genome Res..

[40] Filatov D.A., Moneger F., Negrutiu I., Charlesworth D. (2000). Low variability in a Y-linked plant gene and its implications for Y-chromosome evolution. Nature.

[41] Krasovec M., Chester M., Ridout K., Filatov D.A. (2018). The mutation rate and the age of the sex chromosomes in *Silene latifolia*. Curr. Biol..

[42] Bergero R., Charlesworth D. (2011). Preservation of the Y transcriptome in a 10-million-year-old plant sex chromosome system. Curr. Biol..

[43] Chibalina M.V., Filatov D.A. (2011). Plant Y chromosome degeneration is retarded by haploid purifying selection. Curr. Biol..

[44] Bergero R., Qiu S., Charlesworth D. (2015). Gene loss from a plant sex chromosome system. Curr. Biol..

[45] Muyle A., Zemp N., Fruchard C., Cegan R., Vrana J., Deschamps C., Tavares R., Hobza R., Picard F., Widmer A. (2018). Genomic imprinting mediates dosage compensation in a young plant XY system. Nat. Plants.

[46] Muyle A., Zemp N., Deschamps C., Mousset S., Widmer A., Marais G.A. (2012). Rapid de novo evolution of X chromosome dosage compensation in *Silene latifolia*, a plant with young sex chromosomes. PLoS Biol..

[47] Papadopulos A.S., Chester M., Ridout K., Filatov D.A. (2015). Rapid Y degeneration and dosage compensation in plant sex chromosomes. Proc. Natl. Acad. Sci. USA.

[48] Krasovec M., Kazama Y., Ishii K., Abe T., Filatov D.A. (2019). Immediate dosage compensation is triggered by the deletion of Y-linked genes in *Silene latifolia*. Curr. Biol..

[49] Qiu S., Bergero R., Guirao-Rico S., Campos J.L., Cezard T., Gharbi K., Charlesworth D. (2016). RAD mapping reveals an evolving, polymorphic and fuzzy boundary of a plant pseudoautosomal region. Mol. Ecol..

[50] Qiu S., Bergero R., Charlesworth D. (2013). Testing for the footprint of sexually antagonistic polymorphisms in the pseudoautosomal region of a plant sex chromosome pair. Genetics.

[51] Bergero R., Qiu S., Forrest A., Borthwick H., Charlesworth D. (2013). Expansion of the pseudo-autosomal region and ongoing recombination suppression in the *Silene latifolia* sex chromosomes. Genetics.

[52] Bergero R., Forrest A., Kamau E., Charlesworth D. (2007). Evolutionary strata on the X chromosomes of the dioecious plant *Silene latifolia*: Evidence from new sex-linked genes. Genetics.

[53] Zhang G.J., Li C., Li Q.Y., Li B., Larkin D.M., Lee C., Storz J.F., Antunes A., Greenwold M.J., Meredith R.W. (2014). Comparative genomics reveals insights into avian genome evolution and adaptation. Science.

[54] Li B., Dewey C.N. (2011). RSEM: Accurate transcript quantification from RNA-Seq data with or without a reference genome. BMC Bioinform..

[55] Li H., Handsaker B., Wysoker A., Fennell T., Ruan J., Homer N., Marth G., Abecasis G., Durbin R., Subgroup G.P.D.P. (2009). The sequence alignment/map format and SAMtools. Bioinformatics.

[56] McKenna A., Hanna M., Banks E., Sivachenko A., Cibulskis K., Kernytsky A., Garimella K., Altshuler D., Gabriel S., Daly M. (2010). The Genome Analysis Toolkit: A MapReduce framework for analyzing next-generation DNA sequencing data. Genome Res..

[57] Edgar R.C. (2004). MUSCLE: Multiple sequence alignment with high accuracy and high throughput. Nucleic Acids Res..

[58] Tajima F. (1989). Statistical method for testing the neutral mutation hypothesis by DNA polymorphism. Genetics.

[59] Ramos-Onsins S.E., Ferretti L., Raineri E., Jené J., Marmorini G., Burgos W., Vera G. Mstatspop: Statistical Analysis Using Multiple Populations for Genomic Data. https://bioinformatics.cragenomica.es/numgenomics/people/sebas/software/software.html.

[60] Filatov D.A. (2018). The two “rules of speciation” in species with young sex chromosomes. Mol. Ecol..

[61] Hu X.S., Filatov D.A. (2016). The large-X effect in plants: Increased species divergence and reduced gene flow on the Silene X-chromosome. Mol. Ecol..

[62] Raudsepp T., Chowdhary B.P. (2015). The eutherian pseudoautosomal region. Cytogenet. Genome Res..

[63] Eyre-Walker A. (1993). Recombination and mammalian genome evolution. Proc. Biol. Sci..

[64] Campos J.L., Halligan D.L., Haddrill P.R., Charlesworth B. (2014). The relation between recombination rate and patterns of molecular evolution and variation in *Drosophila melanogaster*. Mol. Biol. Evol..

[65] Charlesworth B., Campos J.L. (2014). The relations between recombination rate and patterns of molecular variation and evolution in Drosophila. Annu. Rev. Genet..

[66] Graves J.A., Wakefield M.J., Toder R. (1998). The origin and evolution of the pseudoautosomal regions of human sex chromosomes. Hum. Mol. Genet..

[67] Rappold G.A. (1993). The pseudoautosomal regions of the human sex chromosomes. Hum. Genet..

[68] Palmer S., Perry J., Kipling D., Ashworth A. (1997). A gene spans the pseudoautosomal boundary in mice. Proc. Natl. Acad. Sci. USA.

[69] White M.A., Ikeda A., Payseur B.A. (2012). A pronounced evolutionary shift of the pseudoautosomal region boundary in house mice. Mamm. Genome.

[70] Raudsepp T., Chowdhary B.P. (2008). The horse pseudoautosomal region (PAR): Characterization and comparison with the human, chimp and mouse PARs. Cytogenet. Genome Res..

[71] Das P.J., Chowdhary B.P., Raudsepp T. (2009). Characterization of the bovine pseudoautosomal region and comparison with sheep, goat, and other mammalian pseudoautosomal regions. Cytogenet. Genome Res..

[72] Kirkpatrick M., Guerrero R.F. (2014). Signatures of sex-antagonistic selection on recombining sex chromosomes. Genetics.

