# Supplementary material for: The Location of the Pseudoautosomal Boundary in Silene latifolia
Source: Genes (Basel). 2020 May 31;11(6):610. doi: 10.3390/genes11060610 (PMC7348893; doi:10.3390/genes11060610)
Supplement: Supplementary file 1 [file genes-11-00610-s001.zip › KrasovecEtAl_suppl/tableS1.pdf]

Table S1. Genotype of wild females at SNPs inheritd from father to sons in the df108 family
Y-alleles in females are highlighted with green background

gene name	SNP pos	Y-allele	X-allele	genotype in females				
				fSa526b	fSa615	fSa758d	fSa833d.	fSa985
Contig697	1131	T	C	C/C	C/C	C/C	C/C	C/C
Contig697	1257	A	G	G/G	G/G	G/G	G/G	G/G
Contig697	1268	T	C	C/C	C/C	C/C	C/C	C/C
Contig697	1269	G	A	A/A	A/A	A/A	A/A	A/A
Contig697	1797	C	T	T/T	T/T	T/T	T/T	T/T
Contig15301	240	A	G	G/G	G/G	G/G	G/G	G/G
Contig12513	459	C	T	T/T	T/T	T/T	T/T	T/T
Contig12513	489	T	G	G/G	G/G	G/G	G/G	G/G
Contig12513	492	T	C	C/C	C/C	C/C	C/C	C/C
Contig14349	318	C	T	T/T	T/T	T/T	T/T	T/T
Contig14349	319	A	C	C/C	C/C	C/C	C/C	C/C
Contig14349	334	A	G	G/G	G/G	G/G	G/G	G/G
Contig14349	337	G	T	T/T	T/T	T/T	T/T	T/T
Contig14349	349	C	T	T/T	T/T	T/T	T/T	T/T
Contig14349	351	T	A	A/A	A/A	A/A	A/A	A/A
Contig14349	445	C	T	T/T	T/T	T/T	T/T	T/T
Contig14349	1009	C	T	T/T	T/T	T/T	T/T	T/T
Contig14349	1023	C	A	A/A	A/A	A/A	A/A	A/A
Contig14349	1126	T	G	G/G	G/G	G/G	G/G	G/G
Contig14349	1142	T	G	G/G	G/G	G/G	G/G	G/G
Contig14349	1150	A	G	G/G	G/G	G/G	G/G	G/G
Contig14349	1169	C	A	A/A	A/A	A/A	A/A	A/A
Contig14349	1225	A	G	G/G	G/G	G/G	G/G	G/G
Contig14349	1264	T	G	G/G	G/G	G/G	G/G	G/G
Contig14349	1271	G	A	A/A	A/A	A/A	A/A	A/A
Contig14349	1301	T	C	C/C	C/C	C/C	C/C	C/C
Contig17773	203	T	C	C/C	C/C	C/C	C/C	C/C
Contig17773	251	A	G	G/G	G/G	G/G	G/G	G/G
Contig17773	350	G	C	C/C	C/C	C/C	C/C	C/C
Contig17773	398	G	A	A/A	A/A	A/A	A/A	A/A
Contig17773	424	C	G	G/G	G/G	G/G	G/G	G/G
Contig17773	435	T	C	C/C	C/C	C/C	C/C	C/C
Contig17773	446	T	C	C/C	C/C	C/C	C/C	C/C
Contig17773	485	G	A	A/A	A/A	A/A	A/A	A/A
Contig17773	524	T	C	C/C	C/C	C/C	C/C	C/C
Contig17773	553	G	C	C/C	C/C	C/C	C/C	C/C
Contig17773	569	T	G	G/G	G/G	G/G	G/G	G/G
Contig17773	582	A	G	G/G	G/G	G/G	G/G	G/G

Contig17773	620	G	T	T/T	T/T	T/T	T/T	T/T
Contig17773	689	G	A	A/A	A/A	A/A	A/A	A/A
Contig17773	761	T	A	A/A	A/A	A/A	A/A	A/A
Contig17773	854	A	T	T/T	T/T	T/T	T/T	T/T
Contig17773	923	C	T	T/T	T/T	T/T	T/T	T/T
Contig17773	1046	A	T	T/T	T/T	T/T	T/T	T/T
Contig17773	1102	G	A	A/A	A/A	A/A	A/A	A/A
Contig17773	1163	T	C	C/C	C/C	C/C	C/C	C/C
Contig17773	1169	A	G	G/G	G/G	G/G	G/G	G/G
Contig17773	1211	T	C	C/C	C/C	C/C	C/C	C/C
Contig17773	1257	T	C	C/C	C/C	C/C	C/C	C/C
Contig17773	1261	T	C	C/C	C/C	C/C	C/C	C/C
Contig17773	1274	T	G	G/G	G/G	G/G	G/G	G/G
Contig17773	1336	T	A	A/A	A/A	A/A	A/A	A/A
Contig17773	1356	C	A	A/A	A/A	A/A	A/A	A/A
Contig8598	362	C	T	T/T	T/T	T/T	T/T	T/T
Contig8598	370	A	C	C/C	C/C	C/C	C/C	C/C
Contig8598	374	T	C	C/C	C/C	C/C	C/C	C/C
Contig8598	375	T	C	C/C	C/C	C/C	C/C	C/C
Contig8598	380	G	A	A/A	A/A	A/A	A/A	A/A
Contig8598	410	C	T	T/T	T/T	T/T	T/T	T/T
Contig8598	413	A	C	C/C	C/C	C/C	C/C	C/C
Contig8598	425	G	A	A/A	A/A	A/A	A/A	A/A
Contig8598	426	T	C	C/C	C/C	C/C	C/C	C/C
Contig8598	716	T	C	C/C	C/C	C/C	C/C	C/C
Contig8598	1079	C	T	T/T	T/T	T/T	T/T	T/T
Contig1798	1444	T	C	C/C	C/C	C/C	C/C	C/C
Contig1798	1450	T	C	C/C	C/C	C/C	C/C	C/C
Contig1798	1461	A	G	G/G	G/G	G/G	G/G	G/G
Contig1798	1465	C	A	A/A	A/A	C/A	A/A	A/A
Contig9505	47	A	G	G/G	G/G	G/G	G/G	G/G
Contig9505	79	T	C	T/C	T/C	T/C	C/C	C/C
Contig9505	93	T	C	C/C	C/C	C/C	C/C	C/C
Contig9505	154	T	G	G/G	G/G	G/G	G/G	G/G
Contig9505	231	T	A	A/A	A/A	A/A	A/A	A/A
Contig9505	632	A	G	G/G	G/G	G/G	G/G	G/G
Contig9505	696	G	A	A/A	A/A	A/A	A/A	A/A
Contig9505	777	T	G	G/G	G/G	G/G	G/G	G/G
Contig9505	1030	G	A	A/A	A/A	A/A	A/A	A/A
Contig9505	1035	T	A	A/A	A/A	A/A	A/A	A/A
Contig9505	1098	C	T	T/T	T/T	T/T	T/T	T/T
Contig9505	1121	T	A	A/A	A/A	A/A	A/A	A/A

Contig9505	1284	A	G	G/G	G/G	G/G	G/G	G/G
Contig9505	1481	T	C	C/C	C/C	C/C	C/C	C/C
Contig18786	648	T	C	C/C	C/C	C/C	C/C	C/C
Contig18786	649	T	C	C/C	C/C	C/C	C/C	C/C
Contig2117	965	C	T	T/T	T/T	T/T	T/T	T/T
Contig1046	76	G	T	T/T	T/T	T/T	T/T	T/T
Contig1046	134	T	G	G/G	G/G	G/G	G/G	G/G
Contig1046	137	G	C	C/C	C/C	C/C	C/C	C/C
Contig1046	227	T	G	G/G	T/G	T/G	G/G	G/G
Contig1046	439	G	A	A/A	G/A	A/A	A/A	A/A
Contig1046	451	A	G	A/G	A/G	G/G	G/G	G/G
Contig1046	510	G	A	G/A	G/A	A/A	A/A	A/A
Contig1046	760	C	T	T/T	T/T	T/T	T/T	T/T
Contig1046	770	G	T	T/T	T/T	T/T	T/T	T/T
Contig1046	910	A	G	G/G	G/G	G/G	G/G	G/G
Contig1046	1239	T	C	C/C	C/C	C/C	C/C	C/C
Contig1046	1579	G	C	C/C	C/C	C/C	C/C	C/C
Contig1046	1591	G	T	T/T	T/T	T/T	T/T	T/T
Contig1251	137	T	A	T/A	T/A	T/A	A/A	A/A
Contig1251	180	A	G	G/G	G/G	G/G	G/G	G/G
Contig1251	340	A	C	C/C	C/C	C/C	C/C	C/C
Contig1251	777	A	T	T/T	T/T	T/T	T/T	T/T
Contig1251	814	A	G	G/G	G/G	G/G	G/G	G/G
Contig1251	969	G	C	C/C	C/C	G/C	C/C	C/C
Contig1251	1189	A	G	G/G	G/G	G/G	G/G	G/G
Contig1251	1192	G	A	A/A	A/A	A/A	A/A	A/A
Contig1251	1222	G	A	A/A	A/A	A/A	A/A	A/A
Contig1251	1298	G	T	T/T	T/T	T/T	T/T	T/T
Contig1251	1384	C	T	T/T	T/T	T/T	T/T	T/T
Contig1251	1408	A	G	G/G	G/G	G/G	G/G	G/G
Contig1251	1411	G	T	T/T	T/T	T/T	T/T	T/T
Contig1251	1447	G	A	A/A	A/A	A/A	A/A	A/A
Contig1251	1507	C	T	C/T	C/T	T/T	T/T	T/T
Contig1251	1517	A	G	G/G	G/G	G/G	G/G	G/G
Contig1251	1540	A	G	A/G	G/G	G/G	G/G	G/G
Contig1251	1588	A	G	G/G	G/G	G/G	G/G	G/G
Contig1251	1663	A	C	C/C	C/C	C/C	C/C	C/C
Contig1251	1669	G	A	A/A	A/A	A/A	A/A	A/A
Contig1251	1672	G	A	A/A	A/A	A/A	A/A	A/A
Contig1251	1675	G	A	A/A	A/A	A/A	A/A	A/A
Contig1251	1797	A	G	G/G	G/G	G/G	G/G	G/G
Contig1251	1814	T	A	A/A	A/A	A/A	A/A	A/A

Contig13504	243	A	G	G/G	G/G	G/G	G/G	G/G
Contig13504	426	A	G	G/G	G/G	G/G	G/G	G/G
Contig13504	469	T	C	C/C	C/C	C/C	C/C	C/C
Contig13504	516	A	C	C/C	C/C	C/C	C/C	C/C
Contig13504	894	A	G	G/G	G/G	G/G	G/G	G/G
Contig1623	671	G	A	A/A	A/A	A/A	A/A	A/A
Contig1623	674	G	T	T/T	T/T	T/T	T/T	T/T
Contig1623	917	C	A	A/A	A/A	A/A	A/A	A/A
Contig528	147	C	T	T/T	T/T	T/T	T/T	T/T
Contig528	164	G	C	C/C	C/C	C/C	C/C	C/C
Contig528	644	G	T	T/T	T/T	T/T	T/T	T/T
Contig528	653	C	T	T/T	T/T	T/T	T/T	T/T
Contig528	848	T	G	G/G	G/G	G/G	G/G	G/G
Contig528	884	T	C	C/C	C/C	C/C	C/C	C/C
Contig528	927	A	G	G/G	G/G	G/G	G/G	G/G
Contig528	1025	T	C	C/C	C/C	C/C	C/C	C/C
Contig528	1052	T	G	G/G	G/G	G/G	G/G	G/G
Contig528	1352	T	C	C/C	C/C	C/C	C/C	C/C
Contig528	1657	C	G	G/G	G/G	G/G	G/G	G/G
Contig13419	63	T	G	G/G	G/G	G/G	G/G	G/G
Contig13419	109	C	G	G/G	C/G	G/G	G/G	G/G
Contig13419	110	A	C	C/C	A/C	C/C	C/C	C/C
Contig13419	159	A	G	G/G	G/G	G/G	G/G	G/G
Contig13419	160	A	C	C/C	C/C	C/C	C/C	C/C
Contig13419	261	A	G	G/G	G/G	G/G	G/G	G/G
Contig13419	278	C	T	T/T	T/T	T/T	T/T	T/T
Contig13419	311	G	C	C/C	C/C	C/C	C/C	C/C
Contig13419	364	C	A	A/A	A/A	A/A	A/A	A/A
Contig13419	396	C	T	T/T	T/T	C/T	T/T	T/T
Contig13419	551	A	G	G/G	G/G	G/G	G/G	G/G
Contig13419	592	A	C	C/C	C/C	C/C	C/C	C/C
Contig13419	1371	C	T	T/T	T/T	T/T	T/T	T/T
Contig13419	1374	T	C	C/C	C/C	C/C	C/C	C/C
Contig13419	1378	A	G	G/G	G/G	G/G	G/G	G/G
Contig13419	1410	G	A	A/A	A/A	A/A	A/A	A/A
Contig13419	1594	T	G	G/G	G/G	G/G	G/G	G/G
Contig13419	1599	T	C	C/C	C/C	C/C	C/C	C/C
Contig13419	1639	C	T	T/T	T/T	C/T	T/T	T/T
Contig13419	1651	G	A	A/A	A/A	A/A	A/A	A/A
Contig13419	1676	C	A	A/A	A/A	A/A	A/A	A/A
Contig13419	1749	C	T	T/T	T/T	T/T	T/T	T/T
Contig13419	1763	T	C	C/C	C/C	C/C	C/C	C/C

Contig15757	283	A	G	G/G	G/G	G/G	G/G	G/G
Contig15757	300	C	T	T/T	T/T	T/T	T/T	T/T
Contig15757	334	G	A	A/A	A/A	A/A	A/A	A/A
Contig15757	366	C	A	A/A	A/A	A/A	A/A	A/A
Contig15757	406	A	T	T/T	T/T	T/T	T/T	T/T
Contig15757	425	A	T	T/T	T/T	T/T	T/T	T/T
Contig15757	537	G	A	A/A	A/A	A/A	A/A	A/A
Contig15757	539	T	C	C/C	C/C	C/C	C/C	C/C
Contig15757	702	T	C	C/C	C/C	C/C	C/C	C/C
Contig15757	705	G	A	A/A	A/A	A/A	A/A	A/A
Contig15757	711	T	G	G/G	G/G	G/G	G/G	G/G
Contig15757	725	T	C	C/C	C/C	C/C	C/C	C/C
Contig15757	732	C	T	T/T	T/T	T/T	T/T	T/T
Contig15519	519	T	G	G/G	G/G	G/G	G/G	G/G
Contig15519	1132	T	C	C/C	C/C	C/C	C/C	C/C
Contig9011	486	C	T	C/T	C/T	C/T	T/T	T/T
