# Supplementary material for: The Location of the Pseudoautosomal Boundary in Silene latifolia
Source: Genes (Basel). 2020 May 31;11(6):610. doi: 10.3390/genes11060610 (PMC7348893; doi:10.3390/genes11060610)
Supplement: Supplementary file 1 [file genes-11-00610-s001.zip › KrasovecEtAl_suppl/tableS2.pdf]

Table S2. Location of genes adjacent to PAR-boundary in the *S. latifolia* genomic scaffolds published in ref [47]. The scafolds containing >1 gene are shown in bold.

Gene				Genomic scaffold		%	Pos. in scaffold	
#	cDNA	map pos	PAR?	name	length	identity	start	end
1	Contig675	62.6	X	QBIE01001963.1	371,899	99.73	26,286	34,629
2	Contig697	62.6	X	QBIE01030564.1	8,309	99.27	3,502	7,879
3	Contig804	62.6	X	QBIE01000326.1	49,350	100.00	15,301	26,640
4	Contig867	62.6	X	QBIE01041289.1	8,602	100.00	73	7,460
5	Contig8509	62.6	X	QBIE01003146.1	25,308	99.06	7,192	24,889
6	Contig8598	62.6	X	QBIE01004030.1	68,990	98.45	10,741	19,409
7	Contig8660	62.6	X	QBIE01001396.1	44,622	99.32	44,029	44,471
8	Contig12513	62.6	X	QBIE01018774.1	13,172	100.00	6,441	10,995
9	Contig15301	62.6	X	QBIE01001029.1	89,827	99.70	38,483	38,810
10	Contig1564	62.6	X	QBIE01031006.1	10,699	98.91	217	2,802
11	Contig1740	62.6	X	QBIE01001590.1	90,538	99.85	75,360	76,034
12	Contig17645	62.6	X	QBIE01000680.1	93,542	99.80	72,262	73,770
13	Contig18491	62.6	X	QBIE01144983.1	1,447	100.00	1,176	1,447
14	Contig1290	62.6	X	QBIE01011154.1	16,893	99.64	4,608	15,121
15	Contig14349	62.6	X	QBIE01002388.1	24,242	98.71	16,380	19,502
16	Contig1436	62.6	X	QBIE01011960.1	24,831	100.00	7,258	23,580
17	Contig15401	62.6	X	QBIE01013336.1	14,174	99.77	1,555	2,872
18	Contig17773	62.6	X	QBIE01010323.1	14,859	100.00	949	14,767
19	Contig1804	62.6	X	QBIE01000292.1	26,470	100.00	8,110	13,415
20	Contig18911	62.6	X	QBIE01017539.1	9,926	99.79	8,154	8,638
21	Contig2431	62.6	X	QBIE01010644.1	21,309	98.84	2,333	5,379
22	Contig2761	62.6	X	QBIE01017837.1	16,917	100.00	9,535	10,775
23	Contig2802	62.6	X	QBIE01000937.1	11,560	100.00	8,533	8,849
24	Contig3835	62.6	X	QBIE01005015.1	5,732	100.00	214	1,487
25	Contig3846	62.6	X	QBIE01009689.1	18,878	100.00	5,346	8,424
26	Contig4210	62.6	X	QBIE01019453.1	22,311	100.00	681	2,170
27	Contig4518	62.6	X	QBIE01015231.1	19,373	100.00	16,481	18,191
28	Contig5724	62.6	X	QBIE01004766.1	13,815	98.28	2,108	9,654
29	Contig8488	62.6	X	QBIE01004664.1	63,618	99.84	9,888	17,210
30	Contig1798	62.7	fuzzyB	QBIE01001315.1	25,370	100.00	7,566	24,910
31	Contig9505	62.7	fuzzyB	QBIE01003253.1	2,953	99.18	86	449
32	Contig18786	62.7	fuzzyB	QBIE01000061.1	129,341	99.84	115,905	116,525
33	Contig255	62.7	fuzzyB	QBIE01013388.1	15,062	99.54	7,850	10,195
34	Contig12476	62.8	fuzzyB	QBIE01000862.1	7,785	100.00	1,812	7,760
35	Contig2117	62.9	fuzzyB	QBIE01022098.1	15,253	99.73	3,974	12,150
36	Contig1858	62.9	fuzzyB	QBIE01018657.1	7,401	100.00	4,209	4,893

37	Contig456	62.9	fuzzyB	QBIE01000920.1	69,497	100.00	40,188	46,000
38	Contig1229	63	fuzzyB	QBIE01015569.1	17,696	100.00	3,249	14,524
39	Contig6406	63	fuzzyB	QBIE01002286.1	19,925	99.14	6,691	14,876
40	Contig1046	63.4	fuzzyB	QBIE01004665.1	17,278	98.67	11,375	13,291
41	Contig1251	63.4	fuzzyB	QBIE01012692.1	18,013	98.36	7,978	8,832
42	Contig13504	63.4	fuzzyB	QBIE01001457.1	48,934	97.76	36,166	38,765
43	Contig1623	63.4	fuzzyB	QBIE01019676.1	3,352	98.72	2,493	3,352
44	Contig528	63.4	fuzzyB	QBIE01011343.1	7,229	98.86	6,249	6,598
45	Contig13419	63.6	fuzzyB	QBIE01001873.1	5,748	99.79	1	5,748
46	Contig15757	63.6	fuzzyB	QBIE01021457.1	9,899	100.00	7,087	7,921
47	Contig15519	63.6	fuzzyB	QBIE01011576.1	17,344	100.00	3,289	5,712
48	Contig9011	63.6	fuzzyB	QBIE01001981.1	50,151	100.00	22	2,184
49	Contig16617	64.8	PAR	QBIE01003988.1	108,991	98.32	43,241	46,052
50	Contig8746	65.5	PAR	QBIE01000961.1	87,498	99.28	52,055	66,298
51	Contig634	65.5	PAR	QBIE01009822.1	34,241	100.00	2,361	27,281
52	Contig17188	65.5	PAR	NA				
53	Contig2083	65.7	PAR	QBIE01001139.1	79,114	98.94	22,310	28,782
54	Contig16105	65.7	PAR	QBIE01005794.1	57,368	98.60	40,736	42,360
55	Contig4536	66.3	PAR	NA				
56	Contig15998	66.3	PAR	NA				
57	Contig8522	66.6	PAR	QBIE01009416.1	28,088	98.09	22,658	23,181
58	Contig6681	66.6	PAR	NA				
59	Contig15667	66.7	PAR	QBIE01001925.1	53,183	100.00	6,161	6,722
60	Contig984	66.8	PAR	QBIE01000425.1	64,130	98.25	30,823	53,326
61	Contig8437	66.8	PAR	QBIE01009445.1	35,060	99.18	7,448	9,405
62	Contig1800	66.8	PAR	QBIE01020164.1	9,313	99.43	1,594	1,767
63	Contig1431	66.8	PAR	NA				
64	Contig1089	66.8	PAR	**QBIE01001489.1**	60,031	100.00	27,618	31,565
65	Contig7074	66.9	PAR	NA				
66	Contig1252	66.9	PAR	QBIE01017794.1	11,043	97.73	1,799	2,371
67	Contig8534	66.9	PAR	QBIE01005133.1	33,928	98.21	590	4,216
68	Contig889	66.9	PAR	QBIE01004434.1	100,419	98.32	64,649	93,792
69	Contig1714	66.9	PAR	QBIE01004836.1	36,703	98.53	5,017	8,447
70	Contig1093	67	PAR	NA				
71	Contig924	67	PAR	QBIE01135924.1	5,496	99.17	2,577	5,496
72	Contig1960	67.4	PAR	**QBIE01001489.1**	60,031	99.29	44,209	58,775
73	Contig17553	67.4	PAR	QBIE01000761.1	71,010	100.00	41,525	44,171
74	Contig16348	67.4	PAR	QBIE01037568.1	5,387	99.34	5,086	5,387
75	Contig2235	67.4	PAR	NA				
76	Contig682	70.2	PAR	**QBIE01000100.1**	121,065	99.24	57,082	59,379

77	Contig4687	70.2	PAR	NA				
78	Contig8471	70.2	PAR	QBIE01077958.1	3,659	100.00	63	2,859
79	Contig3881	70.2	PAR	NA				
80	Contig1751	70.2	PAR	**QBIE01000100.1**	121,065	99.13	26,804	34,132
81	Contig2223	71.5	PAR	**QBIE01000100.1**	121,065	98.77	7,817	12,956
82	Contig947	71.7	PAR	QBIE01167309.1	1,197	99.37	1,040	1,197
83	Contig8300	71.9	PAR	QBIE01002687.1	45,138	98.23	13,382	19,328
84	Contig15701	72	PAR	QBIE01003402.1	62,544	100.00	37,034	39,788
85	Contig991	72	PAR	**QBIE01000100.1**	121,065	98.67	50,890	51,039
86	Contig3920	72.1	PAR	QBIE01006256.1	34,860	100.00	13,134	13,240
